# An Intelligent Identification and Repair Method for Annular Holes in 3D Printing

**DOI:** 10.1155/2022/3990216

**Published:** 2022-05-05

**Authors:** Shanhui Zhang, Wei Wu, Wei Wei

**Affiliations:** ^1^School of Control Science and Engineering, Shandong University, Jinan, Shandong 250061, China; ^2^Shandong Shanda Hoteam Software Co., Ltd., Jinan, Shandong 250101, China

## Abstract

With the popularization of 3D printing in the consumer goods field, there is a specific type of hole named annular hole in the narrow features, surface bumps, or folded parts of products. The traditional triangular mesh repair method is not effective for such holes. The structural characteristics of the annular hole are analyzed in this research, and the definition and identification method for annular holes is presented through the shape and position relationship of two closed hole lines. To improve the repair efficiency and the applicability of the algorithm, the traditional hole repair method and process are ameliorated. A repair strategy of hole boundary stitching and filling, triangulation optimization, implicit surface construction based on Radial Basis Function, and surface integral deformation is adopted to achieve a smooth joint of the repaired hole surface with the original triangular mesh surface. The method can ensure surface smoothness and accuracy. Finally, two experiments are carried out to verify the repair quality and efficiency of our method. Compared with Geomagic software, the proposed method can automatically identify and repair annular holes with fewer defects and similar efficiency. Compared with a traditional hole repair method, the evaluation results demonstrate that the proposed method is much faster, and the repair quality is higher without the influences of human operations. It is shown that our method can be applied to annular hole repair of 3D printing models without the participation of technicians.

## 1. Introduction

3D printing is an emerging manufacturing technology based on digital models, which manufactures physical items by stacked bondable materials such as powdered metals or plastics. Having made a profound impact on the traditional process, production line, factory model, and industry chain combination, 3D printing is a representative disruptive technology in manufacturing [1–3]. Currently, with the rapid development and popularity of 3D printing, consumers have also turned to and experienced 3D printing. For example, the platform created by Shapeways in the United States, which integrates design, customization, and sales, has more than 21,000 online stores, having served more than 1 million consumers and printed over 21 million products [4]; in China, Haier Group has built an online open innovation and entrepreneurship platform, which has more than 300,000 registered users and created successful cases such as the Tianzun air conditioner [5].

As 3D printing is applied in the consumer goods field, various types of holes often occur in constructing and acquiring mesh models, which would seriously affect the appearance and quality of 3D printed models. At present, the identification and repair of holes in triangular mesh models are mostly carried out through user interaction, which has low efficiency and requires certain skills of operators. However, most consumers do not have professional skills in hole identification and repair, which poses difficulties for the development of 3D printing in the consumer goods field. The repair of holes like simple holes and island holes has been focused on and researched, but the repair of specific annular holes in the consumer goods field has not been reasonably solved. To this end, it is necessary to research and classify the characteristics of annular holes according to their topology, and corresponding annular hole identification and repair methods could be provided with high repair quality and less professional operation requirement.

The paper is organized as follows. After reviewing some related work in Section 2, the main types and characteristics of annular holes are studied and an intelligent identification method for annular holes is proposed in Section 3. In Section 4, an improved implicit surface repair method for annular holes based on Radial Basis Function (RBF) is presented. In Section 5, compared with the results of internationally well-known commercial software and a traditional hole repair method, experimental results and discussion are given. Finally, our summary, evaluation, and direction for further research are discussed in Section 6.

## 2. Related Work

**Figure 1:**
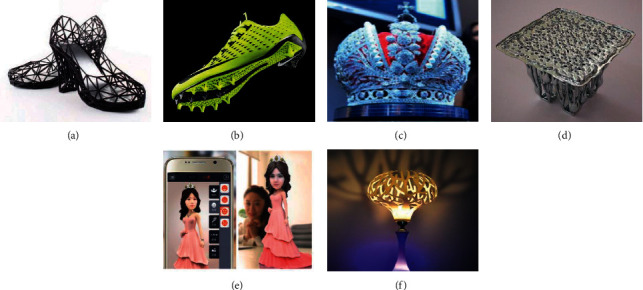
3D printed consumer goods [8–10]. (a) 3D printed shoes STRVCT. (b) Nike 3D printed football shoes. (c) Faux Russian big crown. (d) 3D printed table. (e) Custom doll. (f) Flower lampstand.

Originating as early as the 1980s, 3D printing has boomed again with the promotion of the Internet since 2010. Having made positive progress on the market application in fields including aerospace, automotive, and healthcare, it got started in 3D printed cars, airplanes, and artificial liver tissues. It can be seen that 3D printing is continuing to promote and expand its application. In 2012, the British Magazine the Economist evaluated 3D printing as “an important symbol of the third industrial revolution,” which would become a new creative technology to change the future world [6]. Since then, 3D printing has also gained the attention of the average consumer. In October 2013, the world's first successful auction of a 3D printed artwork named “ONO God” was held [7]. 3D printed shoes, jewelry, dolls, and other products have also gradually entered the vision of ordinary consumers, as shown in Figure 1.

Along with the wide application of 3D printing, many scholars at home and abroad have researched the rapid reconstruction and repair of 3D printed models. The simple and flexible structure, high stability, and topology of triangular mesh models lead to the rapid and mature development of the hole-filling technique based on triangular mesh models [11]. A more typical repair method is the surface-based hole-filling algorithm, which repairs holes on the surface with the help of data near the holes by directly detecting and identifying the hole information of the input 3D model. It mainly includes the mesh growth method based on adding new sampling points and the method based on implicit surface fitting [12–14].

The mesh growth method based on adding new sampling points generates new sampling points from hole triangle boundaries to hole area and constructs new triangular patches by edge swapping and triangle refining until the whole hole is covered. Pernot et al. [15] proposed the curvature minimization principle to repair holes by adding new triangles under the premise of minimizing the curvature variation between the surrounding and inserted meshes, which is applicable to holes with a simple shape. Marchandise et al. [16] used the hole boundary points as well as the neighboring vertices as the basis for establishing surface patches and sampling on the surface patches. The method had a relatively high hole-filling accuracy, but it was not applicable to the case where the curvature variation of the hole area was large. Wang et al. [17] first repaired the hole with the advancing front method and then used the data such as normals of the boundary vertices to determine optimal vertices, so as to adjust the position of the prerepaired hole triangular surface patch. However, for complex holes, especially for the hole boundary area with large curvature variation, the geometric features of the original model would be lost, and the repair result would be too flat.

The hole-filling algorithm based on implicit surface fitting repairs holes with an implicit surface established with original model data. Lévy [18] parametrized the entire model data to the plane for processing, and the algorithm is less efficient when the area of the repaired holes is small. and the number of triangles is large. Brunton et al. [19] first flattened the hole boundaries without self-intersection onto the reference plane for filling and then used the minimum energy method to embed the patched mesh back into the spatial mesh. Fortes et al. [20] proposed a shape feature determination method on top of Brunton's. Based on the minimization of an energy function, the holes were filled by inheriting the local information of the holes in the original model. The method required better local data quality of holes. Du et al. [21–25] all used RBF to fit the implicit surface of holes and then adjusted new vertices to the implicit surface. The method was suitable for repairing simple holes and island holes. For island holes with large curvature variation, a bridging method was proposed to connect the island boundary and hole boundary. Zhao et al. [26] carried out a preliminary repair with the advancing front method and then finished the repair by solving Poisson equation to adjust positions of added vertices. This method was not applicable to the repair of large holes.

Through the above analysis on hole-filling techniques, it is proved that the mesh growth-based repair method is a simple idea, easy to implement, and more effective for repairing holes in flat areas with simple structures and no significant features. The implicit surface fitting method produces a smoother repair surface, inherits the original mesh data information during the repair process, and has little interference with the original mesh model and more accurate repair results. However, it asks for a harsher scenario and would always smooth sharp features. Both methods can obtain ideal results for simple holes, but there may be mapping failure or mapping error for complex holes, resulting in poor hole-filling effects.

For the segmentation and repair of complex holes, Jun [27] proposed an algorithm to split a complex hole into simple holes and then fill each divided simple hole, which helped in triangulating and repairing holes with self-intersecting boundaries in the projection process but was not ideal in filling complex holes of other types, especially the problem of self-intersecting boundaries in the nonprojection process. Inspired by this method, Li et al. [28] presented an algorithm based on the concept of edge expansion to split complex holes into simple holes and then perform hole-filling with due consideration of the neighboring mesh morphology. Lai and Hsu [29] further considered the treatment of island holes and proposed a hole-filling algorithm based on B-sample surfaces to fit the vertices near the holes to the B-sample surfaces, emphasizing the topological accuracy and smoothness of the joint between the new mesh and the existing mesh. Feng et al. [30] introduced a fast filling method for triangular meshes based on hole size, which classified holes into small-sized, middle-sized, and large-sized holes according to their size and used different filling algorithms for different hole types. However, the classification method, which only considered the hole size and ignored the complex topological morphology, would hardly achieve satisfactory results in filling complex holes. Wen et al. [31] presented a surface-repairing method with automatic identification of defective holes and maintenance of detail features in the hole region, but the method was only applicable to repair defective holes in simply connected domains, ignoring the topology of complex missing regions. By analyzing the hole boundaries and topology, Li [12] classified holes into ordinary holes, interstitial voids, island holes, and semiclosed holes and proposed a hole-filling algorithm based on Poisson equation, which can repair large-scale complex 3D models containing a large number of holes and a variety of hole types. The method was applied to the restoration of heritage models with slightly insufficient repair efficiency. Centin et al. [32] proposed a Poisson-driven approach, which allowed to close complex holes, islands, gaps, and missing parts with a seamless integration of the patching triangles along the mesh boundaries, with guaranteed and homogeneous mesh quality. But its repair quality was seriously influenced by the boundary curves user selected. Based on the above analysis, the intelligent identification and repair method for annular holes is still a challenge.

## 3. Annular Holes and Their Intelligent Identification Method

### 3.1. Generation of Annular Holes

**Figure 2:**
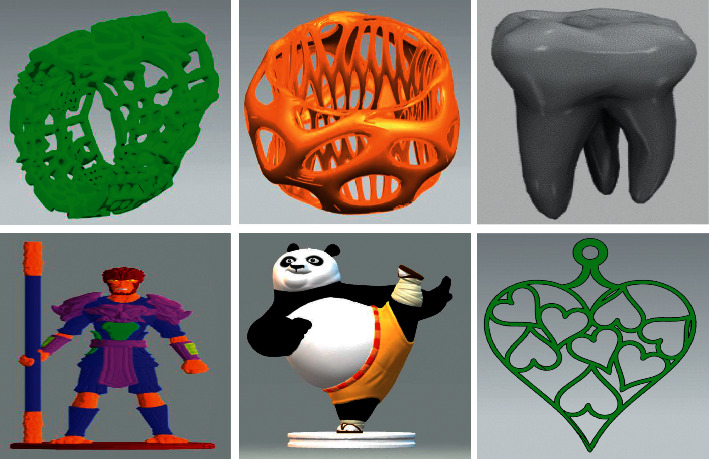
The example products prone to annular holes.

In the consumer goods field, due to the complex and varied structures of most products such as handicrafts, doll models, and bionic products, there are many narrow features, surface bulges, hollow-carved structures, or folds, as shown in Figure 2. They can easily cause fracture, local loss, or separation of the triangular mesh model during model construction, scanning, and acquisition. So, a specific type of hole always occurs, namely, an annular hole. Using the existing repair methods, it is easy to cause the fillings of hollow-carved structures or folds, and they lead to high differences from the original structure. In addition, most users for consumer goods are ordinary people lacking the ability to operate specialist repair software, so the result of hole repair is not satisfactory.

### 3.2. Annular Holes

**Figure 3:**
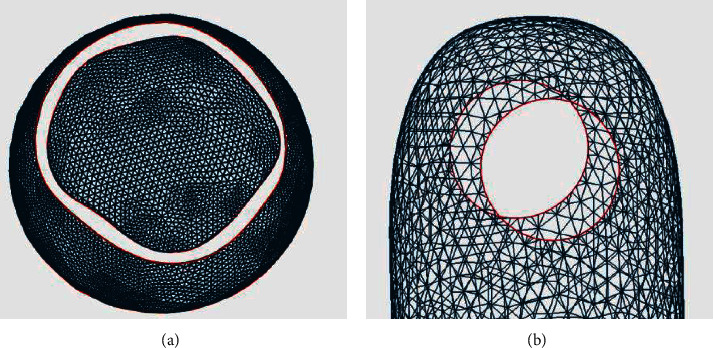
Two types of annular holes. (a) Coplanar annular hole. (b) Parallel annular hole.

The annular hole is formed by two boundary lines with similar shape and no common point, whose positional relationship is approximate coplanar or parallel, as shown in Figure 3. Annular holes are commonly found in sections, cross-sections, or folds of models. Two types of annular holes are summarized through analysis.

Coplanar annular hole: the positions of two annular closed curves are approximately coplanar, and the normal directions are similar, as shown in Figure 3(a). It is a common type of annular holes occurred in the surface bulges or hollow-carved structures.

Parallel annular hole: the positions of two annular closed curves are approximately parallel, and the normal directions are nearly contrary, as shown in Figure 3(b). It is an uncommon type of annular holes occurred in narrow features, hole features, and folds. There are few repair methods to deal with such holes.

The annular hole has two annular closed curves. The application of simple hole filling methods to an annular hole will repair all the interior of the two annular closed curves, resulting in a large number of self-intersecting patches. As for island hole repair methods, the number of internal triangular surface patches of annular holes is too large, and the hole normal directions of parallel annular holes are nearly contrary. So, it is not appropriate to simply use the surface deformation repair method of deleting island fragments or selecting some islands as constraint points, which is easy to affect the repair quality or destroy the original structure of the model. For this reason, the repair of annular holes needs to be treated separately.

### 3.3. Intelligent Identification Method for Annular Holes

As the annular hole is made up of two boundaries that are similarly located and shaped, the hole connects two discontinuous triangular mesh sections of the model. The two boundaries of the annular hole can be identified by detecting the distance between the boundaries of different combinations of triangular facets; that is, an annular hole is composed of the free boundaries of two sets of triangular meshes. Therefore, the following determination method for annular holes is presented.

We set the hole boundary points as *P*_*1*_*, P*_*2*_*, P*_*3*_,…, *P*_*n*_, and *O* as the gravity center of hole boundary points, i.e.,

(1) $$\begin{array}{c} O\left(\bar{X},\bar{Y},\bar{Z}\right)=\frac{1}{n}\sum_{i=1}^{n}P_{i}，\;i=1,2,\ldots ,n. \end{array}$$

Using the coordinates of the hole boundary points and the gravity center *O*, a 3 × 3 covariance matrix *C* can be defined as

(2) $$\begin{array}{c} C=\left[\begin{array}{ccc} \text{cov}\left(X,X\right) & \text{cov}\left(X,Y\right) & \text{cov}\left(X,Z\right)\\ \text{cov}\left(Y,X\right) & \text{cov}\left(Y,Y\right) & \text{cov}\left(Y,Z\right)\\ \text{cov}\left(Z,X\right) & \text{cov}\left(Z,Y\right) & \text{cov}\left(Z,Z\right) \end{array}\right], \end{array}$$

where

(3) $$\begin{array}{c} \mathrm{cov}\left(X,Y\right)=\frac{\sum_{i=1}^{n}\left(X_{i}-\bar{X}\right)\left(Y_{i}-\bar{Y}\right)}{n-1}. \end{array}$$

The eigenvalues and eigenvectors of the matrix can be calculated through Jacobi method. We set the three eigenvalues as the first vector $\overset{⟶}{V_{1}}$ and the eigenvector corresponding to the smallest eigenvalue as the second vector $\overset{⟶}{V_{2}}$. The eigenvalues and eigenvectors of the Jacobi matrix represent the distribution direction of the points, and the main feature that distinguishes an annular hole from an island hole is the approximation of the distribution pattern of the points between two boundaries. Therefore, the characteristic distance between two boundaries of the annular hole is defined as

(4) $$\begin{array}{c} Dis_{ab}=\overset{⟶}{V_{a1}}\times \overset{⟶}{V_{b1}}+\overset{⟶}{V_{a2}}\times \overset{⟶}{V_{b2}}. \end{array}$$

**Figure 4:**
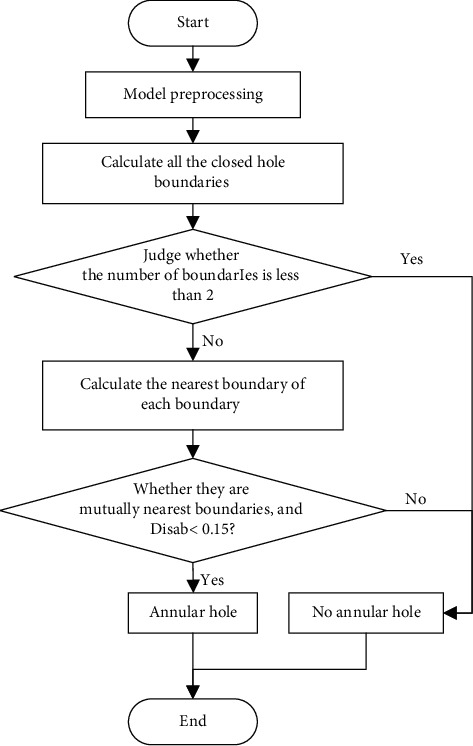
Intelligent identification process for annular holes.

In this equation, *a* and *b* represent the two boundaries of an annular hole. *Dis*_*ab*_ is the characteristic distance between boundaries *a* and *b*. $\overset{⟶}{V_{a1}}$ and $\overset{⟶}{V_{b1}}$ are the first vectors of boundaries *a* and *b*. $\overset{⟶}{V_{a2}}$ and $\overset{⟶}{V_{b2}}$ are the second vectors of boundaries *a* and *b*. Then, we screen a large number of annular holes and island holes and calculate their characteristic distances. An empirical value of the characteristic distance is found to distinguish an annular hole from an island hole. When *Dis*_*ab*_ < 0.15, an annular hole is formed between the two boundaries. At this point, the corresponding repair method can be used to achieve optimal results. Accordingly, an intelligent identification process for annular holes is planned as in Figure 4.

**Step 1 .**

Model preprocessing. Segment all the complex holes to single holes without common points.

**Step 2 .**

Calculate all the closed hole boundaries, and exclude crack holes and dislocation holes. The method in Steps 1 and 2 has been researched in another thesis [33].

**Step 3 .**

Judge whether the number of closed boundaries is less than 2. If yes, this indicates that there is no annular hole, and it ends; if no, proceed to next step.

**Step 4 .**

Calculate the nearest boundary of each boundary. It is measured by the distance between the nearest points between two boundaries.

**Step 5 .**

**Figure 5:**
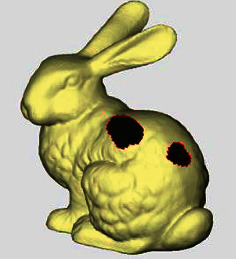
Example of adjacent simple holes.

Judge whether they are mutually nearest boundaries, and the boundary feature distance is less than 0.15. Mutual nearest boundary means that the nearest boundary of boundary *a* is *b*, and the nearest boundary of boundary *b* is *a*. At the same time, in order to eliminate the case that two simple holes are adjacent (as shown in Figure 5), it is also necessary to meet the following conditions: on the fitting plane of the boundary, the gravity center of boundary *a* is within *b*, and the gravity center of boundary *b* is within *a*. If the above conditions are met, it is an annular hole. Otherwise, there is no annular hole.

**Step 6 .**

End.

## 4. Repair Method for Annular Holes

Traditional hole-filling methods generally require a process of surface fitting, surface cutting, and surface stitching. The annular hole has two boundaries and two sets of corresponding cutting and stitching boundaries, with narrow or flat hole lines and massive boundary points. Using traditional hole-filling methods, it always has low repair efficiency and a high failure rate. Besides, various consumer products lead to a variety of annular holes, and the systematicness and robustness of the above method are also insufficient. Therefore, we propose a method that stitches and fills the holes, optimizes the triangulation, constructs implicit surface, and finally carries out surface integral deformation. The method can solve the repair issue of diversified annular holes and extend the application range in consumer products.

### 4.1. Hole Boundary Stitching and Filling

**Figure 6:**
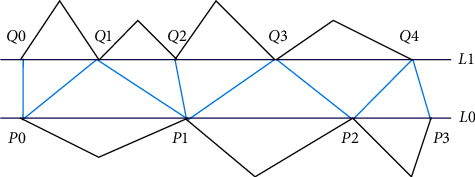
Stitch annular hole boundaries.

First of all, an initial stitching surface patch is constructed as the basis for surface fitting. In this paper, the method of connecting the nearest points is used to stitch annular holes, as shown in Figure 6. The specific steps are described as follows.

**Step 7 .**

Reorient hole lines. As the two types of annular holes have different hole line directions, the two hole lines of an annular hole need to be adjusted to the same direction. The same direction refers to the fact that, on the best-fit plane of the two hole lines, the polygons formed by the projection of the hole lines are all clockwise or counterclockwise. Herein, the counterclockwise direction is taken as positive.

**Step 8 .**

Connect initial triangles. Start from the starting point *P*_0_ of the hole line *L*_0_ with fewer points, calculate the nearest point *Q*_0_ of *P*_0_ on the other hole line *L*_1_, and connect *P*_0_ and *Q*_0_. Find the next point *Q*_1_ of *L*_1_ and its nearest point *Q*_2_ on *L*_1_, and connect *P*_1_ and *Q*_2_. Triangulate the area composed of *P*_0_-*P*_1_-*Q*_2_-*Q*_0_ to obtain triangles *P*_0_*Q*_1_*Q*_0_, *P*_0_*P*_1_*Q*_1_, and *P*_1_*Q*_2_*Q*_1_.

**Step 9 .**

Connect the remaining triangles. Repeat the above steps for the points on *L*_0_ until all boundary points on *L*_0_ are calculated.

### 4.2. Triangulation Optimization

**Figure 7:**
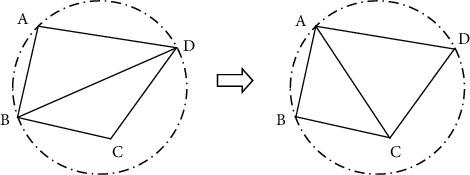
Illustration of the edge-swap method.

Before the surface is fitted, the stitched triangle patches need to be refined, so that it can be smoothly connected to the original model. The specific optimization principle and method are as follows: (1) for a triangular patch with an area larger than the specified area, divide the patch into three triangular patches by taking a point at the center of the triangle and connecting that point to its three vertices. (2) If two triangles are connected by an edge, and one triangle is inside the circumscribed circle of the other triangle, the edge is called a long and narrow edge. The edge-swap method should be used for optimization. Edge-swap is achieved by swapping the diagonals of the convex quadrilateral formed by two adjacent triangles, so that the narrow triangle converges to a positive triangle, as shown in Figure 7.

### 4.3. Implicit Surface Construction

The initial repair surface has been created by stitch and refined triangulation of the two hole lines of the annular hole, but it is not yet smoothly integrated with the whole surface. For that, the RBF is used to establish an implicit surface equation for annular holes, to ensure as much continuity and smoothness as possible between the repaired surface and the original surface.

Firstly, to ensure a smooth and continuous joint between the hole boundary and its surrounding original triangular surface, we use the vertex of the two boundaries of an annular hole and the multiplet neighboring points of its neighboring triangular patches (typically 3–5 multiplet neighboring points, depending on the size of the hole) to build a collection *V*={*P*_*i*_, *i*=1,2,…, *n*} (*n* is the number of hole boundary vertices and neighboring points) of interpolation constraint points. The interpolation constraint points on the surface satisfy

(5) $$\begin{array}{c} f\left(P_{i}\right)=0,\;i=1,2,\ldots ,n. \end{array}$$

In order to avoid useless solutions of *f* ≡ 0, while keeping the positions of the boundary points of stitched patches unchanged, we calculate the normal information of the refined mesh model boundaries and add additional constraint points in the normal direction. They are located in the inner or outer directions of the surface. The additional constraint points should satisfy

(6) $$\begin{array}{c} f\left(P_{i}+h_{i}N_{i}\right)=h_{i}, \end{array}$$

where *N*_*i*_ is the normal vector of the surface located at the vertex *P*_*i*_, and*h*_*i*_ is a normal constraint value with a small positive value. It can be assumed that all additional constraint points are located at the same distance from the surface, and they are on an equivalent surface to the hole surface. The value of the constraint does not affect the solution of the implicit equation for the hole surface, so the implicit equation for the hole surface can be taken to have a value of *h*_*i*_=1 at all additional constraint points.

From this, we combine all interpolated constraint points and additional constraint points to create a set of constraint points *V*={*P*_*i*_, *i*=1,2,…, *N*} (N is the number of all the hole constraint points) and form the following constraint:

(7) $$\begin{array}{c} f\left(P_{i}\right)=h_{i},\;i=1,2,\ldots ,N. \end{array}$$

From the constraint points, we can define the implicit surface equation *f*(**r**)=0. The energy function [22, 34] for a thin plate with its second-order differentiable function is

(8) $$\begin{array}{c} E=\int_{R^{3}}\left(\frac{\partial^{2}f}{\partial x^{2}}+\frac{\partial^{2}f}{\partial y^{2}}+\frac{\partial^{2}f}{\partial z^{2}}+2\frac{\partial^{2}f}{\partial x\partial y}+2\frac{\partial^{2}f}{\partial x\partial z}+2\frac{\partial^{2}f}{\partial y\partial z}\right)\text{d}x\text{d}y\text{d}z. \end{array}$$

This energy function reflects the smoothness of the function *f* in three dimensions, and it has lower energy values in regions of the surface where there are no sharp changes in curvature such as folds. The interpolation function is solved under the interpolation constraint of *f*(*P*_*i*_)=*h*_*i*_ such that the value of the energy function is minimized. At this point, the form of the interpolation function *f* is obtained as the RBF form [13]:

(9) $$\begin{array}{c} f\left(r\right)=\sum_{j=1}^{N}w_{j}\phi \left(r-P_{j}\right)+Q\left(r\right). \end{array}$$

In this equation, **r** denotes any point on the generated surface, **r**=(*x*, *y*, *z*); *P*_*j*_ denotes the points defining the equation, *P*_*j*_=(*P*_*j*_^*x*^, *P*_*j*_^*y*^, *P*_*j*_^*z*^); *w*_*j*_ denotes the real weight corresponding to each constraint point; *Q*(*r*) is a first order polynomial. For any point ***r***, the form of *Q*(*r*) is *Q*(*r*)=*q*_0_+*q*_1_*x*+*q*_2_*y*+*q*_3_*z*, where *q*_0_, *q*_1_, *q*_2_ and *q*_3_ are real coefficients of the polynomial. *ϕ*(*r* − *P*_*j*_) is the RBF. In three-dimensional space, as functions with three variables need to be fitted, the more effective form of RBF is a triharmonic spline function *ϕ*(**r**)=**r**^3^.

In order to solve for the weights and polynomial coefficients, each constraint point must satisfy both the interpolation constraint and the orthogonality conditions.

(10) $$\begin{array}{c} f\left(P_{i}\right)=\sum_{j=1}^{N}w_{j}\phi \left(P_{i}-P_{j}\right)+Q\left(P_{i}\right),\\ \sum_{j=1}^{N}w_{j}=\sum_{j=1}^{N}w_{j}P_{j}^{x}=\sum_{j=1}^{N}w_{j}P_{j}^{y}=\sum_{j=1}^{N}w_{j}P_{j}^{z}=0. \end{array}$$

Let *ϕ*_*ij*_=*ϕ*(*P*_*i*_ − *P*_*j*_). From the above conditions, we obtain the following set of linear equations:

(11) $$\begin{array}{c} \left[\begin{array}{cccccccc} \phi_{11} & \phi_{12} & \cdots & \phi_{1n} & 1 & p_{1}^{x} & p_{1}^{y} & p_{1}^{z}\\ \phi_{21} & \phi_{22} & \cdots & \phi_{2n} & 1 & p_{2}^{x} & p_{2}^{y} & p_{2}^{z}\\ \vdots & \vdots & \ddots & \vdots & \vdots & \vdots & \vdots & \vdots \\ \phi_{N1} & \phi_{N2} & \cdots & \phi_{NN} & 1 & p_{N}^{x} & p_{N}^{y} & p_{N}^{z}\\ 1 & 1 & \cdots & 1 & 0 & 0 & \cdots & 0\\ p_{1}^{x} & p_{2}^{x} & \cdots & p_{N}^{x} & 0 & 0 & \cdots & 0\\ p_{1}^{y} & p_{2}^{y} & \cdots & p_{N}^{y} & 0 & 0 & \cdots & 0\\ p_{1}^{z} & p_{2}^{z} & \cdots & p_{N}^{z} & 0 & 0 & \cdots & 0 \end{array}\right]\left[\begin{array}{c} w_{1}\\ w_{2}\\ \vdots \\ w_{N}\\ q_{0}\\ q_{1}\\ q_{2}\\ q_{3} \end{array}\right]=\left[\begin{array}{c} h_{1}\\ h_{2}\\ \vdots \\ h_{N}\\ 0\\ 0\\ 0\\ 0 \end{array}\right]. \end{array}$$

For the annular hole repair surface, as the constraint points taken are data points around the hole boundary, the solution time is shorter with the Gauss elimination method, and a unique set of solutions (*w*_1_, *w*_2_,…*w*_*N*_, *q*_0_, *q*_1_, *q*_2_, *q*_3_) can be obtained directly. Bring the obtained results into the interpolation function *f*(**r**), and the implicit surface equation for the hole established by the RBF can be given as

(12) $$\begin{array}{c} f\left(x,y,z\right)=\sum_{j=1}^{n}w_{j}{\left(\sqrt{{\left(x-P_{j}^{x}\right)}^{2}+{\left(y-P_{j}^{y}\right)}^{2}+{\left(z-P_{j}^{z}\right)}^{2}}\right)}^{3}+q_{0}+q_{1}x+q_{2}y+q_{3}z=0. \end{array}$$

### 4.4. Surface Integral Deformation

Once the implicit equation for the annular hole surface has been established, the vertices of triangular patches from the triangulation optimization in 4.2 need to be adjusted to the fitted implicit surface, that is, integral deformation of the surface. In this paper, the classical gradient descent method is used to adjust the vertices of triangular patches after triangulation, where all the repaired vertices of triangular patches are gradually approximated towards the implicit surface along the gradient direction of the implicit equation *f*(*x*, *y*, *z*), until they are adjusted to the fitted hole implicit surface within the allowed error range.

Gradient descent is an iterative optimization-seeking algorithm for some criterion function. Let *f*(*r*) be some criterion function and ***r*** a vector, and then the negative gradient direction of ***r*** is the direction, where *f*(*r*) decreases fastest, along which the approximation point can be reached fastest [35]. The gradient of the implicit function *f*(*x*, *y*, *z*) is denoted as ∇*f*=(∂*f*/∂*x*, ∂*f*/∂*y*, ∂*f*/∂*z*) [22]. From the implicit surface equation for holes, we get

(13) $$\begin{array}{c} \frac{\partial f}{\partial x}=3\sum_{j=1}^{N}w_{j}\left(x-P_{j}^{x}\right)\sqrt{{\left(x-P_{j}^{x}\right)}^{2}+{\left(y-P_{j}^{y}\right)}^{2}+{\left(z-P_{j}^{z}\right)}^{2}}+q_{1},\\ \frac{\partial f}{\partial y}=3\sum_{j=1}^{N}w_{j}\left(y-P_{j}^{y}\right)\sqrt{{\left(x-P_{j}^{x}\right)}^{2}+{\left(y-P_{j}^{y}\right)}^{2}+{\left(z-P_{j}^{z}\right)}^{2}}+q_{2},\\ \frac{\partial f}{\partial z}=3\sum_{j=1}^{N}w_{j}\left(z-P_{j}^{z}\right)\sqrt{{\left(x-P_{j}^{x}\right)}^{2}+{\left(y-P_{j}^{y}\right)}^{2}+{\left(z-P_{j}^{z}\right)}^{2}}+q_{3}. \end{array}$$

Depending on the vertex of each triangulated patch, the new position to which it is adjusted needs to be calculated. Considering the iteration efficiency and the optimization effect, we chose the following equation as the iteration formula:

(14) $$\begin{array}{c} r_{k+1}=r_{k}-\frac{f\left(r_{k}\right)}{\left\|\nabla f{\left(r_{k}\right)}^{2}\right\|}\nabla f\left(r_{k}\right), \end{array}$$

where *k* denotes the number of iterations. Calculate the difference *r*_*k*+1_ − *r*_*k*_ between the new position of the vertex and the position before stretching. If *r*_*k*+1_ − *r*_*k*_ ≤ *ε* (*ε* is a given limited error), it can be considered that the new position of the vertex *r*_*k*+1_ is on the hole surface and the iteration ends. Otherwise, *r*_*k*+1_ replaces *r*_*k*_ and the iteration continues.

The implicit surface equation of the hole describes the surface formed by the hole boundaries and their multiplet neighboring triangular patches, so the vertices of the adjusted triangles are located on the same surface as the vertices of the triangular patches around the hole boundary. It is ensured that the repaired hole surface is well stitched to the original triangular mesh surface and maintains a consistent surface form and continuity.

## 5. Experimental Results and Discussion

As few scholars have researched automatic identification and repair methods for annular holes, it is difficult to find a targeted algorithm for comparison. For this reason, this paper selects and compares the repair results with the well-known commercial software Geomagic and a traditional hole repair method to determine the identification and repair effect of the research method. All the developed algorithms were implemented in C++ by using Visual Studio 2013 and tested on a PC equipped with an Intel®Core i7-4790 processor and 8 GB of RAM on Windows 10. In the comparison process, three types of consumer-oriented models are selected, such as shoe, bone, and tooth, because these models are prone to the presence of annular holes.

**Figure 8:**
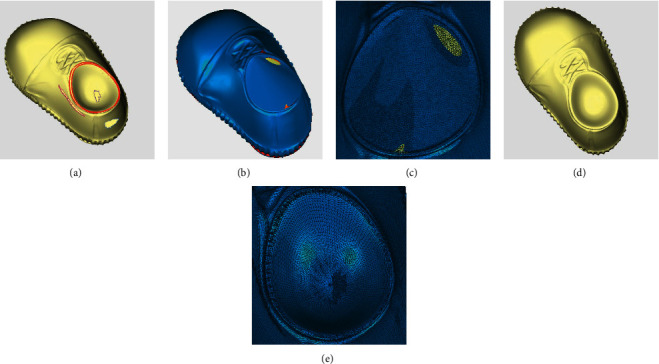
Comparison of repair effect of shoe model. (a) Original annular hole model. (b) Repair result of Geomagic. (c) Repair details of Geomagic. (d) Repair results of the proposed method in this paper. (e) Repair details of the proposed method in this paper.

Figure 8 compares the repair effect of shoe model between Geomagic and the proposed method.  Figure 8(a) shows that an annular hole exists at the folding location of the shoe mouth, and the annular hole lines are indicated by red lines. When using Geomagic to repair, the two hole lines of the shoe model are repaired, respectively, resulting in the closure of the shoe mouth, and the repaired surface differs greatly from that of the original model, shown in Figures 8(b) and 8(c). With the proposed method, the annular hole of the shoe model is accurately identified and repaired between the hole boundaries, as shown in Figure 8(d). The repair details are displayed in Figure 8(e). It can be seen that triangular meshes are evenly divided, and the surface fitted between the two separated surfaces is smooth.

**Figure 9:**
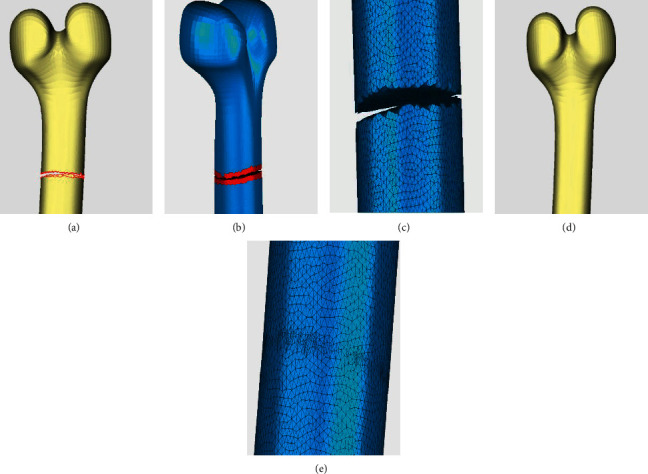
Comparison of repair effect of bone model. (a) Original annular hole model. (b) Repair result of Geomagic. (c) Repair details of Geomagic. (d) Repair results of the proposed method in this paper. (e) Repair details of the proposed method in this paper.

Figure 9 shows the comparison of repair effect of bone model. An annular hole exists at the fracture location of the bone, as shown in Figure 9(a), and the annular hole lines are also indicated by red lines. Geomagic repairs the broken holes, respectively, resulting in the formation of two independent parts, as Figures 9(b) and 9(c) depict. With the proposed method, the annular hole of the bone model is accurately identified and repaired, leading to regular triangular meshes and smooth surfaces, as shown in Figures 9(d) and 9(e).

**Figure 10:**
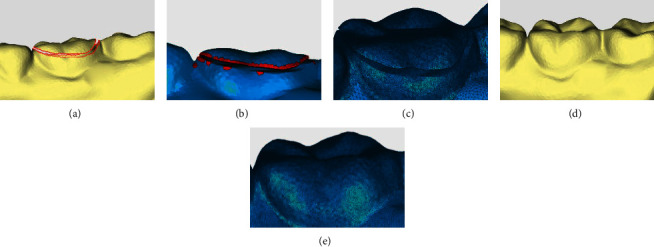
Comparison of repair effect of tooth model. (a) Original annular hole model. (b) Repair result of Geomagic. (c) Repair details of Geomagic. (d) Repair results of the proposed method in this paper. (e) Repair details of the proposed method in this paper.

When repairing the annular hole on the tooth surface, the result is similar to the shoe and bone models, as shown in Figure 10. Through the above cases, we can draw the following conclusions. Geomagic cannot automatically identify annular holes. Without human intervention, the software fills the inside of two closed hole boundaries, leading to discrepancies between the restoration and the actual requirements, and even to the separation of the model. Geomagic destroys the original structure of the model. With the proposed method, annular holes in the model are all accurately identified and repaired between the hole boundaries. As can be observed through the detailed graphs, the structures of the repaired model match the original models, and the triangular mesh density, the continuity with the edge meshes, and the smoothness are all ideal.

After repairing by the proposed method and Geomagic, the three repaired models were checked by a grid doctor. In terms of the six types of repair defects including nonmanifold edges, self-intersections, highly-creased edges, spikes, small components, and small holes, Geomagic is found to have more defects like spikes, highly creased edges, and self-intersections, while the model repaired by the proposed method has fewer defects and higher repair quality. The repair time of our method is equivalent to Geomagic, so the repair efficiency is acceptable. Specific comparison information is shown in Table 1.

**Figure 11:**
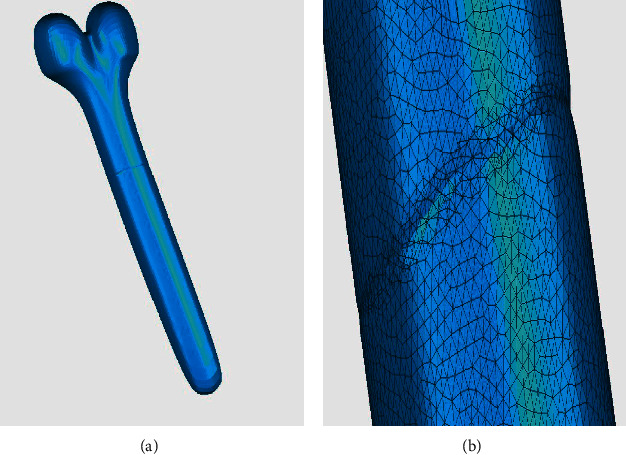
Repair result of bone model with the method proposed by Centin. (a) The overall repair result of bone model with the method proposed by Centin. (b) Repair details of bone model with the method proposed by Centin.

The existing repair methods, when applied to the repair of annular holes, mostly require manual involvement. Taking the algorithms proposed by Centin [32] as an example, the user input for their method must be a set of boundary curves without defects, and holes or gaps, which are not selected should not be filled. So, the repair quality is seriously affected by the input boundaries. For the bone model, we selected boundaries with slight defects to repair. An obvious dent defect appeared, as shown in Figure 11(a). With their restricted Delaunay triangulation and tailoring routine, there is a high quality mesh in the repair area, as shown in Figure 11(b). But the repair time was significantly increased to 1.278 s without considering time spent on the manual selection of boundary lines, and our time was 0.062 s with the automatic identification of boundary lines. Therefore, our method has the advantage of high repair efficiency.

In summary, as repair algorithms or software like Geomagic cannot independently identify and process annular holes, manual repair method is needed. The use of manual repair requires the selection of two closed hole boundary curves, complex manual bridging, and splitting of annular holes into ordinary holes. The result and quality are affected by the skills of technicians, and the efficiency is drastically reduced, which also makes the operation difficult for average consumer users. Therefore, the above comparison shows that the proposed method can automatically determine whether two closed hole boundary lines form an annular hole by their shape and distance relationships and provide intelligent repair methods of stitching and filling, triangulation optimization, and surface integral deformation. The method can meet the repair needs of various types of consumer-oriented models, reduce the operational difficulties of nonspecialist technicians, and improve repair efficiency.

## 6. Conclusion

In the field of consumer goods such as handicrafts and bionic products, narrow features, surface bulges, and folds can easily lead to annular holes in the triangular mesh model during the process of construction, scanning, and acquisition. However, the traditional repair method for common holes is unsuitable for annular holes, which would cause defects such as self-intersecting patches, or even alteration of the original structure of the model.

To this end, we propose an intelligent identification and repair algorithm for annular holes. From the structural characteristics of the annular hole, we give the definition and the automatic identification method for annular holes through analysis of the shape and position relationships between the two closed hole lines. Referring to the traditional implicit surface repair method of single hole, we propose a new improved method. In order to optimize the systematicness and robustness of the repair method, we first stitch and fill the two annular hole lines, then optimize triangulation, construct a variational implicit surface based on RBF, and finally deform the surface integrally based on gradient descent. The proposed method achieves a smooth joint of the repaired hole surface with the original triangular mesh surface, and the repaired surface is smooth and accurate. Finally, we verify the feasibility of the method by examples, proving that it can replace the manual repair methods and significantly improve the repair quality and efficiency.

There are two important improvements in our method. Firstly, it retains all the facet information in the annular hole and stitches the facet between two closed hole lines, instead of filling a single hole boundary in sequence or using the artificial bridging repair method of technicians. The repair quality is also ensured. Secondly, the routine of stitching and filling, triangulation optimization, and surface integral deformation has higher repair efficiency. Especially for parallel annular holes, it avoids possible stitching errors caused by the inconsistent normal vectors of holes. But the errors always appear in other repair methods or software.

In the repair process, the implicit surface repair method based on RBF can obtain smoother repair surfaces, but it also smooths out sharp features. In the future, we will provide corresponding repair methods for more special features. The narrow features, surface bulges, hollow-carved structures, or folds occurring in consumer products will be identified and repaired more accurately. When the method is applied to the repair of 3D printed models in the consumer goods field, it will not require the participation of technicians. The easy operation characteristics of the method will attract the attention and attempt of a large number of consumers. It will promote the widespread use of 3D printing technology.

## Figures and Tables

**Table 1:** Comparison of repair results after inspection.

Objects	Methods	Nonmanifold edges	Self-intersections	Highly creased edges	Spikes	Small components	Small holes	Repair time (s)
Shoe	Geomagic	0	102	503	453	0	0	0.077
	Method in this paper	0	11	39	136	0	0	0.086

Bone	Geomagic	0	0	0	357	0	0	0.056
	Method in this paper	0	0	0	0	0	0	0.062

Tooth	Geomagic	0	90	62	1398	0	0	0.046
	Method in this paper	0	8	48	479	0	0	0.058

## Data Availability

The data used to support the findings of this study are included within the article.
